# Transcriptional Activity of Human Endogenous Retroviruses in Human Peripheral Blood Mononuclear Cells

**DOI:** 10.1155/2015/164529

**Published:** 2015-02-05

**Authors:** Emanuela Balestrieri, Francesca Pica, Claudia Matteucci, Rossella Zenobi, Roberta Sorrentino, Ayele Argaw-Denboba, Chiara Cipriani, Ilaria Bucci, Paola Sinibaldi-Vallebona

**Affiliations:** ^1^Department of Experimental Medicine and Surgery, University of Rome Tor Vergata, Via Montpellier 1, 00133 Rome, Italy; ^2^Institute of Translational Pharmacology, National Research Council, Via Fosso del Cavaliere 100, 00133 Rome, Italy

## Abstract

Human endogenous retroviruses (HERVs) have been implicated in human physiology and in human pathology. A better knowledge of the retroviral transcriptional activity in the general population and during the life span would greatly help the debate on its pathologic potential. The transcriptional activity of four HERV families (H, K, W, and E) was assessed, by qualitative and quantitative PCR, in PBMCs from 261 individuals aged from 1 to 80 years. Our results show that HERV-H, HERV-K, and HERV-W, but not HERV-E, are transcriptionally active in the test population already in the early childhood. In addition, the transcriptional levels of HERV-H, HERV-K, and HERV-W change significantly during the life span, albeit with distinct patterns. Our results, reinforce the hypothesis of a physiological correlation between HERVs activity and the different stages of life in humans. Studies aiming at identifying the factors, which are responsible for these changes during the individual's life, are still needed. Although the observed phenomena are presumably subjected to great variability, the basal transcriptional activity of each individual, also depending on the different ages of life, must be carefully considered in all the studies involving HERVs as causative agents of disease.

## 1. Background

Retroelements are a major contributor to genome size, constituting approximately 45% of the human genomic DNA [1, 2]. They are subdivided, according to size and functionally related structures, into short interspersed elements (SINEs), long interspersed elements (LINEs), long terminal repeat- (LTR) retrotransposons, and DNA transposons [3]. In the past these sequences were wrongly defined “junk DNA” because they were thought not to possess any physiological role [4] but always more evidences show that they are also subject to natural selection and contribute to the benefits of the host, as well as other genes which make up the genome. It is known, in fact, that retroelements play an important role in modeling the genome by increasing the plasticity and evolution of the network of regulation of gene expression [5].

The major subset of LTR retrotransposons is represented by the Human Endogenous Retroviruses (HERVs), which constitute about 8% of the human genome [1]. These elements have their origin in ancient infections of germ cells by exogenous retroviruses during primate evolution [6] and are inserted as proviruses into the cell's chromosomal DNA. If the integration and the possible replication of the virus do not prevent fertilization, the fetus that is formed carries the retroviral element in all somatic and germ line cells. Thus, the provirus becomes part of the human genome and is transmitted to the following generations [7]. Intact HERV sequences share the canonical structure of retroviruses consisting of an internal region of four essential viral genes (*gag*,* pro*,* pol,* and* env*) [8], flanked by two long terminal repeats (LTRs) elements, having the capacity to exert regulatory influences as promoters and/or enhancers of cellular genes [9, 10]. HERVs are classified into families named according to the specificity of the tRNA primer-binding site and the HERV-K is the most recent acquired provirus. During evolution, HERVs were amplified and spread by repeated events of retrotransposition and/or reinfection, resulting in multiple copies, distributed and fixed in the DNA of all human cells [9, 11].

The accumulation of postinsertional mutations and deletions caused in HERVs the lack of an extracellular phase, rendering them noninfectious [10]. Many HERVs are still exceptionally well preserved and maintain open reading frames encoding functional viral proteins [12]. Among these are the syncytin-1 and syncytin-2 [13, 14], which contribute to the development of the syncytiotrophoblast and tolerance of the mother to the fetus [15].

In addition to their physiological role, HERVs have been proposed as possible cofactors in the aetiology of various diseases [16, 17] including multiple sclerosis [18–20], rheumatoid arthritis [21, 22], systemic lupus erythematosus [23], cancer [11, 24], and neurological disorders [25–28].

The HERV-H, HERV-K, HERV-W, and HERV-E families are the most studied in relation to the onset and/or progression of several human diseases, but others, such as HTLV-related endogenous sequence-1 (HRES-1), have been described as playing a role in the progression of systemic lupus erythematosus (SLE) and as possible marker of the disease [29].

There is a general agreement about the need for further research on HERVs, which has to be performed, however, within a rigorous and robust experimental framework [30] to produce a significant advance in knowledge in the specific domain. At present, only few information about HERVs in the general population is available in literature and this consists of data obtained from small healthy control groups included in studies on HERVs-related diseases.

The aim of the study was to determine the transcriptional activity of HERV-H, HERV-K, HERV-W, and HERV-E in a large sample of human subjects, who were apparently healthy and were aged between 1 and 80 years, to help gather information relevant to the current debate on the pathophysiological role of HERVs.

## 2. Methods

### 2.1. Study Population and Ethic Statement

Our study population consisted of 261 Caucasian individuals (128 females and 133 males), aged from 1 to 80 years, attending the outpatients facilities of the Policlinico Tor Vergata, Rome, Italy, for routine examinations, whose haematological values were all found within the normal reference range. Individuals who were reported to be affected by cancer, autoimmune diseases, infections, acute or chronic inflammatory diseases, or neurological disorders were excluded from the study. The analyses were performed on the leftovers of the laboratory samples from informed individuals and data were gathered anonymously. The study was approved by the Independent Ethical Committee of the University of Rome Tor Vergata (register number 73.11).

### 2.2. Sample Preparation

Peripheral blood mononuclear cells (PBMCs) were separated by density gradient centrifugation (Lympholyte-H, Cedarlane, Hornby, ON, Canada) from heparinized blood of the subjects, according to the standard technique, and immediately analyzed or stored at −80°C until analysis.

### 2.3. Qualitative RT-PCR

The transcriptional activity of four HERV families (HERV-E, HERV-H, HERV-K, and HERV-W), selected on the basis of those more frequently associated with human diseases, was assessed in PBMCs from the subjects included in the study, by qualitative RT-PCR. RNA was isolated using the NucleoSpin RNA kit, according to the manufacturer's instructions (Macherey-Nagel, Düren, Germany), digested with DNase (RQ1 RNase-Free DNase, Promega) for 1 h at 37°C, and quantified by NanoDrop 1000 (Thermo Scientific, DE, USA). 250 ng of DNase-treated RNA was reverse-transcribed into cDNA in a total volume of 25 *μ*L using the High Capacity cDNA Reverse Transcription Kit (Applied Biosystems, Life Technologies, Carlsbad, CA) according to the manufacturer's protocol. The efficacy of DNase treatment was tested amplifying the glyceraldehyde-3-phosphate dehydrogenase gene with specific primers (*GAPDH, GenBank accession number NM_002046, forward primer 5*′*GCTGAGTACGTCGTGGAGTC3*′,* reverse primer 5*′*GGTGGTCCAGGGGTCTTACT3*′) that generate a PCR product of 1219 bp for DNA and of 750 bp for mRNA. Only PCR products of 750 bp were observed (data not shown) to demonstrate the absence of DNA contamination. cDNA was amplified using degenerate primer pairs for HERV-H, HERV-K, HERV-W, and HERV-E, to simultaneously evaluate the transcriptional activity of different virus types belonging to an HERV family [31] or with specific primers for GAPDH, as an internal control (Table 1). No RNA template control reactions were included in all experiments. The PCR products were visualized on 1.5% agarose gels containing 10 *μ*g/mL ethidium bromide (EtBr) in 1x Tris-acetate-EDTA buffer. Samples with detectable transcriptional activity, in which PCR products could be visualized on EtBr-stained agarose gels, were defined as positive, while samples in which no specific band could be detected for any of the tested HERV families, yet positive for the GAPDH housekeeping gene, were defined as negative.

### 2.4. Real-Time PCR

The transcriptional levels of* env* sequences of HERV families were quantitatively assessed in human PBMCs, by real-time PCR, in a Bio-Rad instrument (CFX96 Real-Time System), using SYBR Green chemistry (SYBR Real Green PCR Master Mix, Eppendorf). We selected specific primer pairs (Table 2) for* env* of HERV-H, HERV-K, and HERV-W, as previously described [26]. To set up the real-time reaction a serial dilution (10-fold) was done to calculate efficiencies and correlation coefficient. The amplification efficiency was calculated by formula [efficiency = 10^(−1/slope)^] and all the primer pairs used showed an efficiency ranging from 0.95 to 0.97. Real-time PCR reaction included 0.25 *μ*L of cDNA, 200 nM of each primer, and 12.5 *μ*L of SYBR Real Green PCR Master Mix, in a total volume of 25 *μ*L, and was conducted for 1 cycle at 95°C for 5 min and then for 45 cycles of 95°C for 10 sec and 60°C for 15 sec. Each sample was analysed in triplicate and a negative control (no template reaction) was included in each experiment, to check out any possible contamination. The housekeeping gene *β*-glucuronidase (GUSB) was used to normalize the results (Table 2). Each experiment was completed with a melting curve analysis and all primer pairs showed a single peak in the melting curve analysis, confirming the specificity of amplification and the lack of nonspecific products and primer dimers. Quantification was performed using the threshold cycle (Ct) comparative method. The transcriptional levels were calculated as follows: 2^−[ΔCt(sample)−ΔCt(calibrator)]^ = 2^−ΔΔCt^, where ΔCt (sample) = [Ct (HERVH/W* env*) − Ct (GUSB)] and ΔCt (calibrator) was the mean of ΔCT of all the samples analysed. Real-time PCR results that were represented by box plots, depicting mild (black dot) and extreme outliers (asterisk) for each group, were showed.

### 2.5. Statistical Analysis

The Fisher exact test was used to compare the HERVs' transcriptional activity, as determined by qualitative PCR, in different age groups. The Mann Whitney* U* test was used to compare the HERVs' transcriptional levels obtained by quantitative real-time PCR, in different age groups. To determine any correlation between age and HERVs transcription levels, Spearman's rho correlation coefficient was calculated. Statistical analyses were done using the SPSS software (version 17.0). Statistical significant values were considered when *P* < 0.050.

## 3. Results

### 3.1. HERVs Transcriptional Activity in Human PBMCs

**Figure 1:**
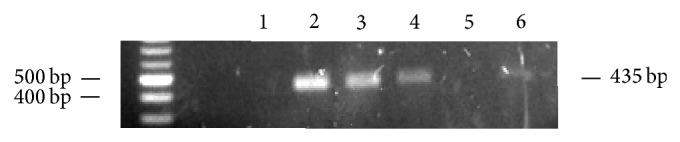
HERV-W transcription activity in human PBMCs, by qualitative RT-PCR. An example of agarose gel analysis is shown. Briefly, RNA from human PBMCs was retrotranscribed and amplified by RT-PCR, using degenerated primers to assess the different virus types belonging to HERV-W family; samples in which PCR products could be visualized were defined as positive for HERV-W expression (lanes 2, 3, 4, and 6), while samples in which no specific band could be detected were defined as negative (lanes 1 and 5).

We examined the transcriptional activity of HERV-H, HERV-K, HERV-W, and HERV-E in PBMCs from 261 subjects, male/female ratio near to 1/1, 1–80 years old, using qualitative RT-PCR (an example of agarose gel analysis of PCR products is shown in Figure 1). The demographic characteristics of the test population are shown in Table 3, where the subjects have been arbitrarily grouped into six different age groups.

The percentages of individuals with detectable transcriptional activity of HERV-H, HERV-K, HERV-W, and HERV-E are reported in Table 4. It can be seen that HERV-H, HERV-K, and HERV-W were all transcriptionally active in the test population, already in the early childhood. In addition, the percentage of samples with detectable transcription activity for HERV-K and HERV-W tended to increase in subjects with more than 40 years of age (*P* < 0.001* versus* all the younger groups, by Fisher exact test). By contrast, HERV-E was transcriptionally active in a very small percentage of individuals.

**Figure 2:**
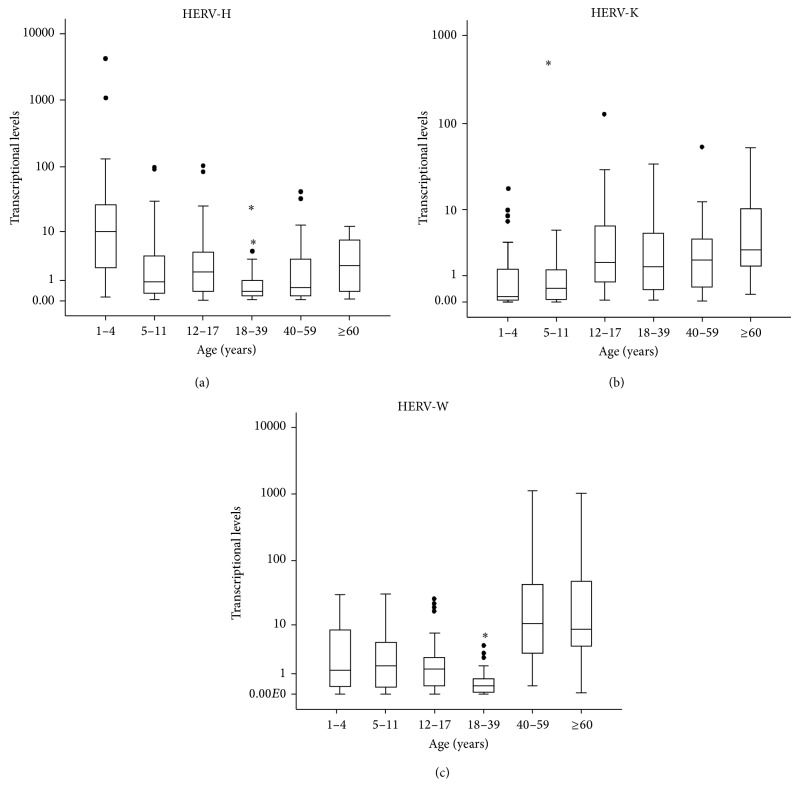
HERV-H, HERV-K, and HERV-W transcriptional levels in human PBMCs. Data are represented as box plot, depicting mild (black dot) and extreme outliers (asterisk). Relative* env* levels were analyzed by real-time PCR and represented by 2^−ΔΔCt^ in logarithmic scale.

We next assessed the levels of transcripts of HERV-H, HERV-K, and HERV-W (looking in particular at the* env* sequences) in PBMCs from the same subjects, by real-time PCR. HERV-E family was excluded from the analysis because of being transcriptionally active only in small percentage of the subjects analysed. The results are shown as box plots, in logarithmic scale in Figure 2 and median values plus interquartile range (IQR) are reported in Table 5.

The highest transcriptional level of HERV-H was found in the youngest subjects (*P* < 0.001, 1–4 years* versus* all the other groups by Mann Whitney* U* test) and the lowest in the group 18–39 years. Indeed, statistically significant differences were found in the comparison of the group 18–39 years with 5–17 years (*P* ≤ 0.033) and with over 60 years (*P* < 0.001).

As it is shown in Figure 2, the transcriptional level of HERV-K found in PBMCs from individuals of both age groups 1–4 and 5–11 years (*P* = 0.465 when compared one each other) was found to be significantly lower than those of all the other groups of individuals (*P* ≤ 0.001).

The transcriptional level of HERV-W (Figure 2) reached the lowest values in the age-group 18–39 years (*P* ≤ 0.001* versus* all the other groups) while it increased significantly in individuals over 40 years (*P* < 0.001 for the comparison with all the other groups).

### 3.2. Correlation of Transcriptional Levels of HERV-H, HERV-K, or HERV-W with the Age of the Test Subjects

The Spearman correlation analysis was used to compare HERV-H, HERV-K, or HERV-W transcriptional levels with the age of the tested subjects. Since both HERV-H and HERV-W showed an age-related bimodal transcriptional activity, with the lowest values being observed in the group 18–39 years, the analysis was performed by dividing the total population into two groups and by using the age median value of the group 18–39, that is, 29.55 years, as a cut-point.

**Figure 3:**
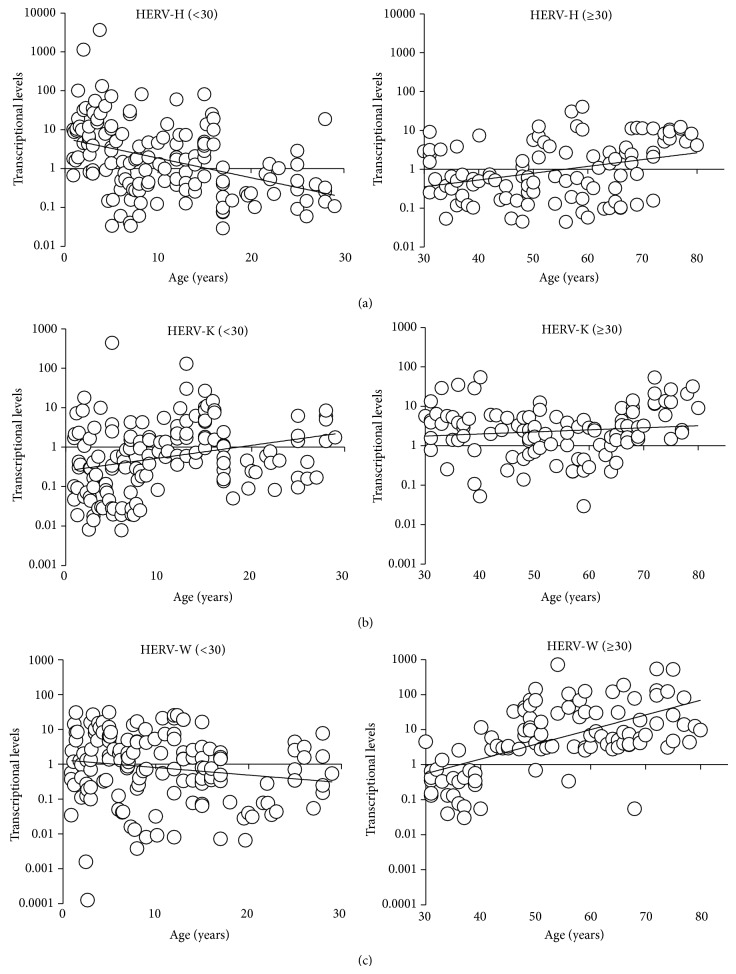
Correlation of HERV-H, HERV-K, or HERV-W* env* levels with the age of the subjects. HERV-H, HERV-K, and HERV-W transcriptional levels are plotted as a function of corresponding age in years. The age median value of the age-group (18–39) was used as a cut-point (left panel: <30; right panel: ≥30). See Results section for correlation Spearman analysis details.* env* levels were analyzed by real-time PCR and represented by 2^−ΔΔCt^ in logarithmic scale.

A negative correlation between the HERV-H transcriptional levels and age was found in individuals <30 years (*rho* = −0.444; *P* < 0.001) (Figure 3, left panel), but a positive correlation was found in individuals ≥30 years (*rho* = 0.337; *P* = 0.001) (Figure 3, right panel). A similar trend, albeit with different rho values, was observed for the HERV-W transcriptional levels (<30 years: rho = −0.195; *P* = 0.018; ≥30 years: rho = 0.573; *P* < 0.001) (Figure 3, right and left panel).

Conversely, a positive correlation between the HERV-K transcription levels and age was found in subjects <30 years (*rho* = 0.307; *P* < 0.001) (Figure 3, left panel), but no significant correlation was found in individuals ≥30 years (*rho* = 0.104; *P* = 0.295) (Figure 3, right panel). Since the HERV-K transcriptional levels increased progressively with age (Figure 2), analysing the total population a stronger value of positive correlation is achieved (*rho* = 0.430; *P* < 0.001). Finally no significant differences were observed in all the statistical tests performed between male and female individuals.

## 4. Discussion

We have investigated the interindividual and the possible age-dependent changes of the HERVs transcriptional activity in human PBMCs, which are the most available cells for human studies [32]. Our results show a different transcriptional activity of the four HERV families analysed with particular age-related changes.

In our test population, the HERV-H, HERV-K, and HERV-W families were found to be transcriptionally active, whereas HERV-E family was far less. In addition, we report that the HERV-H transcription levels share a bimodal trend with the highest levels found in the youngest individuals, the lowest ones in the group 18–39 years, and a further tendency to raise in the older individuals. These findings support the hypothesis that HERV-H plays a role in human development, particularly in the initial phases of life [33, 34].

Also, a positive correlation of the HERV-K transcription levels with age was found in the test population; in fact the levels observed in the younger individuals (≤11 years) were significantly lower than those found in all other age groups. Consistently, HERV-K is known to be one of the most biologically active members of the HERVs families and one of the better responders towards various exogenous stimuli. Since HERV-K family has been associated with different types of cancer [35, 36], the knowledge of a definite and age-related pattern of expression in the general population is relevant for the correlation with the disease.

Similar to HERV-H, also the transcriptional levels of HERV-W family showed a bimodal trend; in fact the levels detected in the youngest individuals were maintained until the age of 17 years, whereas a significant decrease in the group aged 18–39 years and a significant increase in the individuals over 40 years were observed. It is noteworthy that human diseases, which have been associated with an HERV-W overexpression, such as multiple sclerosis and some types of schizophrenia, occur around this range of age [37–39]. Thus, appropriate age-matched healthy controls are absolutely required to avoid bias in the analysis of differences within the groups.

There is a general agreement that the reactivation of HERV-K and HERV-W families is associated with human diseases that develop mostly in adulthood and/or senescence [11, 35, 40] and for which a parallel age-related alteration of DNA methylation is known [41–45]. In fact, HERVs are in the long term epigenetically silenced by DNA methylation and the global DNA methylation levels decline with age in humans [46].

In this regard, it has been demonstrated that monozygotic twins, who are epigenetically indistinguishable during the early years of life, exhibit remarkable differences in the pattern of DNA methylation and histone acetylation, with the increase of age [46, 47]. Moreover, monozygotic twins sharing the greatest epigenetic differences are those who had spent less of their life together, supporting the hypothesis of an accessory role played by the environmental factors [48–50]. Among many factors implied in this phenomenon, a role could be played by the hormones, which can be thus considered as a sort of internal “environmental” factors [51–53]. Since one of the most intriguing characteristics of epigenetics is the reversibility of the induced effects, it is possible to hypothesize that the different hormonal status throughout life may be in part responsible for the different levels of HERVs transcription.

The variations of the transcriptional activity of HERVs during the life span reported in our study reinforce the assumption that phenotypic differences among individuals hinge not only on epigenetic events [44, 46] but also on genetic events [16, 54].

The variability in the expression potential of HERVs at the DNA level is due to defective nature of most HERV proviruses and to the existence of unfixed HERV proviruses that are not present in all human individuals [16, 54]. The presence of polymorphisms provides one explanation of how a ubiquitous gene such as an HERV can cause disease in only a proportion of individuals. An example is provided by polymorphic genotypes of the HRES-1, for which it was described that the relative frequency of genotype I with respect to genotype III was 3.1-fold lower in patients with SLE [55, 56]. High HERV transcription levels cannot be related to disease if they lead to the production of RNA with protective effect, as it has been described for one out of the three HERV-K18 haplotypes, for which a protective effect against the development of type 1 diabetes has been demonstrated in a large family-based association study [57].

The differences between our results and those reported in other studies on HERVs are presumably due to the different experimental approaches, indicating an absolute need to harmonize and to homogenize protocols and techniques, which consider different aspects, such as RNA detection, the functional/regulatory activity of viral proteins, and the copy number variations between individuals and polymorphisms. On the other hand, a possible bias of our study is represented by the not complete characterization of the health status of the tested individuals. In fact, the assessment of the health status was made on the basis of the normal haematological values and of the absence of comorbidities (i.e., cancer, autoimmune diseases, infections, acute or chronic inflammatory diseases, or neurological disorders), as reported by the same individuals. In fact, it is theoretically possible that subclinical variations of the health status might have influenced the HERVs transcriptional activity [58, 59]. However, it is worth mentioning that this is the first study on the domain of four HERVs families RNA transcription, performed in a large sample of individuals with wide age range. Future well-designed prospective studies will greatly help to give reliable answers to the debated question on the physiopathological potential of HERVs.

## 5. Conclusions

To properly assess the causative role of a specific HERV family in a given disease, the direct involvement of determined HERV protein/s in the onset and/or progression of the disease should be demonstrated. At the same time, the comparison of the HERVs activity between patients and controls, matched for age/sex, should be made to avoid misleading results. The present study provides preliminary information about the transcriptional activity of HERV-H, HERV-K, and HERV-W families in human PBMCs. The heterogeneity of the HERVs activity observed is in agreement with data reported in the literature [56–64]. Studies, aiming at identifying the factors, which are responsible for the reported changes during the individual's life, are still needed.

Whether the hypothesis of the physiological correlation between the HERVs transcriptional activity and the stages of development will be further confirmed, the basal transcriptional activity of each individual, also depending on the different ages of life, must be carefully considered in all the studies involving HERVs as causative agents of disease.

## Figures and Tables

**Table 1:** Sequences of degenerated primers used for RT-PCR.

Family	Gene	Forward	Reverse
HERV-E	*gag *	5′-CATCAACCTACTTGGGATTGTCARCA-3′	5′-CAATGACCTTTTTCTTTACAGTAGGCRCA-3′
HERV-H	*gag *	5′-CTTTTATTACCCAATCTGCTCCCGAYAT-3′	5′-TTTAGTGGTGGACAGTCTCTTTTCCARTG-3′
HERV-W	*pol *	5′-GGCCAGGCATCAGCCCAAGACTTG-3′	5′-CTTTAGGGCCTGGAAAGCCACT-3′
HERV-K	*pol *	5′-TCCCCTTGGAATACTCCTGTTTTYGT-3′	5′-CATTCCTTGTGGTAAAACTTTCCAYTG-3′

**Table 2:** Sequences of primers used for real-time PCR.

Family	Gene	Forward	Reverse	GenBank accession number
HERV-H	*env *	5′-TTCACTCCATCCTTGGCTAT-3′	5′-CGTCGAGTATCTACGAGCAAT-3′	AJ289711
HERV-W	*env *	5′-CGTTCCATGTCCCCATTTAG-3′	5′-TCATATCTAAGCCCCGCAAC-3′	NM_014590.3
HERV-K	*env *	5′-CACAACTAAAGAAGCTGACG-3′	5′-CATAGGCCCAGTTGGTATAG-3′	EU308730.1
GUSB		5′-CAGTTCCCTCCAGCTTCAATG-3′	5′-ACCCAGCCGACAAAATGC-3′	NM_000181

**Table 3:** Demographic characteristics of the test population.

Age (years)	Median value (IQR^*^)	Number of samples	Male/female
1–4	2.62 (1.46–3.50)	40	19/21
5–11	7.33 (6.12–8.79)	45	22/23
12–17	15 (13–16)	43	23/20
18–39	31 (25–35)	47	24/23
40–59	50 (48–56)	43	22/21
>60	68 (65–74)	43	23/20

Total		261	133/128

^*^IQR: interquartile range.

**Table 4:** Percentage of individuals with detectable HERVs activity in PBMCs.

Age (years)	Number of samples	HERV-H	HERV-K	HERV-W	HERV-E
1–4	40	65 (26)	80 (32)	65 (26)	5 (2)
5–11	45	91.11 (41)	80 (36)	73.33 (33)	6.67 (3)
12–17	43	88.37 (38)	80 (36)	73.33 (33)	6.98 (3)
18–39	47	70.21 (33)	85.11 (40)	78.72 (37)	4.26 (2)
40–59	43	67.44 (29)	100 (43)	97.67 (42)	6.98 (3)
>60	43	83.72 (36)	100 (43)	95.35 (41)	4.65 (2)

Total	261	76.89 (203)	87.12 (230)	80.30 (212)	5.68 (15)

In parenthesis the number of samples over the total subjects examined is shown.

**Table 5:** Median values and interquartile range of HERV-H, HERV-K, and HERV-W transcriptional levels in human PBMCs.

Age (years)	HERV-H	HERV-K	HERV-W
	Median value (IQR)	Median value (IQR)	Median value (IQR)
1–4	9.89 (1.95–26.66)	0.16 (0.05–1.64)	1.3 (0.27–8.11)
5–11	0.90 (0.28–4.13)	0.43 (0.05–1.33)	1.68 (0.23–5.09)
12–17	1.69 (0.39–4.51)	1.8 (0.64–6.78)	1.38 (0.33–2.62)
18–39	0.38 (0.19–1.07)	1.48 (0.36–5.03)	0.32 (0.07–0.68)
40–59	0.58 (0.18–3.55)	1.98 (0.47–4.28)	10.49 (3.11–42.87)
>60	2.39 (0.32–8.09)	2.89 (1.46–11.37)	8.4 (4.18–76.92)
